# Blue Laser Imaging-Bright Improves Endoscopic Recognition of Superficial Esophageal Squamous Cell Carcinoma

**DOI:** 10.1155/2016/6140854

**Published:** 2016-09-22

**Authors:** Akira Tomie, Osamu Dohi, Nobuaki Yagi, Hiroaki Kitae, Atsushi Majima, Yusuke Horii, Tomoko Kitaichi, Yuriko Onozawa, Kentaro Suzuki, Reiko Kimura-Tsuchiya, Tetsuya Okayama, Naohisa Yoshida, Kazuhiro Kamada, Kazuhiro Katada, Kazuhiko Uchiyama, Takeshi Ishikawa, Tomohisa Takagi, Osamu Handa, Hideyuki Konishi, Yuji Naito, Yoshito Itoh

**Affiliations:** ^1^Department of Molecular Gastroenterology and Hepatology, Graduate School of Medical Science, Kyoto Prefectural University of Medicine, Kyoto, Japan; ^2^Department of Gastroenterology, Murakami Memorial Hospital, Asahi University, Gifu, Japan; ^3^Department of Medical Oncology, Fukushima Medical University, Fukushima, Japan

## Abstract

*Background/Aims*. The aim of this study was to evaluate the endoscopic recognition of esophageal squamous cell carcinoma (ESCC) using four different methods (Olympus white light imaging (O-WLI), Fujifilm white light imaging (F-WLI), narrow band imaging (NBI), and blue laser imaging- (BLI-) bright).* Methods.* We retrospectively analyzed 25 superficial ESCCs that had been examined using the four different methods. Subjective evaluation was provided by three endoscopists as a ranking score (RS) of each image based on the ease of detection of the cancerous area. For the objective evaluation we calculated the color difference scores (CDS) between the cancerous and noncancerous areas with each of the four methods.* Results*. There was no difference between the mean RS of O-WLI and F-WLI. The mean RS of NBI was significantly higher than that of O-WLI and that of BLI-bright was significantly higher than that of F-WLI. Moreover, the mean RS of BLI-bright was significantly higher than that of NBI. Furthermore, in the objective evaluation, the mean CDS of BLI-bright was significantly higher than that of O-WLI, F-WLI, and NBI.* Conclusion*. The recognition of superficial ESCC using BLI-bright was more efficacious than the other methods tested both subjectively and objectively.

## 1. Introduction

Esophageal squamous cell carcinoma (ESCC) is one of the most common causes of cancer death worldwide [1]; more than 10,000 people die from ESCC every year in Japan [2]. The early detection of ESCC leads to an improved prognosis, but when detected at a late stage, ESCC has a poor prognosis [3]. Conventional esophagogastroduodenoscopy with white light imaging (WLI) is frequently used, but it is often difficult to detect superficial ESCCs with this method. Iodine staining is the gold standard for detecting superficial ESCC [4–7] and is based on a chemical reaction between iodine and the glycogen that is contained in the normal epithelial cell microgranules in the stratum spinosum [8]. Dysplastic and cancerous cells are not stained by iodine because they do not contain glycogen due to their lack of cellular maturity. However, iodine staining often induces unpleasant side effects such as severe chest pain and discomfort owing to mucosal irritation [9–11], and its use is not practical in routine endoscopy.

In recent years, image-enhanced endoscopy (IEE) has been reported to improve the detection and diagnosis of superficial ESCC. Narrow band imaging (NBI) has been particularly useful for the detection and diagnosis of superficial ESCC when compared with WLI [12–14]. Blue laser imaging (BLI) is a novel IEE with two different lasers that enable narrow band light observation. BLI is useful to establish the diagnosis of esophageal lesions with or without magnification based on the light-absorption characteristics of hemoglobin (Hb). BLI produces a higher color contrast between the brown esophageal cancer and the surrounding area without magnification [15]. Moreover, BLI-bright is a brighter BLI mode and is useful for endoscopic observation in a distant view. The usefulness of BLI-bright observation in a distant view for colorectal polyps and gastric cancer has already been reported [16, 17]. However, few studies have compared the ability of these different IEEs to detect superficial ESCC. The aim of this study was to evaluate the detection ability of WLI, NBI, and BLI-bright by comparing lesion recognition in still image distant views of ESCC.

## 2. Patients and Methods

### 2.1. Trial Design and Patients

We retrospectively enrolled patients with superficial ESCC who underwent endoscopic submucosal dissection (ESD) at Kyoto Prefectural University of Medicine from March 2012 to December 2014. We selected ESCCs that had endoscopic images obtained at the same angle and distance with NBI and BLI-bright before ESD. Finally, 25 ESCCs with both NBI and BLI-bright recorded images were selected and analyzed in this study. A typical ESCC was observed as a reddish area with the use of Olympus white light imaging (O-WLI) and Fujifilm white light imaging (F-WLI) or as a brownish area with the use of NBI and BLI-bright in a distant view. In all cases, squamous cell carcinoma was histopathologically diagnosed from a biopsy specimen obtained prior to ESD. All patients provided written informed consent for the endoscopic examinations, including the use of NBI and BLI-bright. This study was approved by the ethics committee of Kyoto Prefectural University of Medicine and was registered in the University Hospital Medical Information Network Clinical Trials Registry (UMIN-CTR) as number UMIN000017869.

### 2.2. Endoscopy Systems and IEE Settings

Two different upper GI endoscopic systems manufactured by Olympus Medical Systems Co. and Fujifilm Co. were used in this study. A high-resolution endoscope (GIF-H260Z; Olympus Medical Systems Co., Tokyo, Japan) and a video processor with NBI function (EVIS LUCERA; Olympus Medical Systems Co., Tokyo, Japan) were used for white light imaging (O-WLI) observation and narrow band imaging (NBI) observation. The structure enhancement of the endoscopic video processor was set to B-mode level 3 for O-WLI and B-mode level 8 for NBI. The color mode was fixed at level 1 for NBI.

A high-resolution endoscope (EG-L590ZW; Fujifilm Co., Tokyo, Japan) and a video processor with BLI-bright function (LASEREO; Fujifilm Co., Tokyo, Japan) were used for white light imaging (F-WLI) observation and blue laser imaging-bright (BLI-bright) observation. The structure enhancement of the endoscopic video processor was set to A-mode level 6 for BLI-bright. The color mode was fixed at level C1. The depth of field for the GIF-H260Z and EG-L590ZW endoscope was 7 to 100* * mm and 6 to 100 mm, respectively. The field of view for both endoscopes was 140 degrees.

### 2.3. Endoscopic Procedures

The endoscope was fixed at one angle for observing the lesions, and each lesion was recorded at that fixed angle using O-WLI, F-WLI, NBI, and BLI-bright imaging.

### 2.4. Histopathological Diagnosis

After ESD resection, the lesions were extended and fixed on boards with pins in 20% formalin, and a clinical pathologist histopathologically confirmed the diagnosis of ESCC according to the Japanese Classification of Esophageal Carcinomas.

### 2.5. Subjective Evaluation

**Figure 1:**
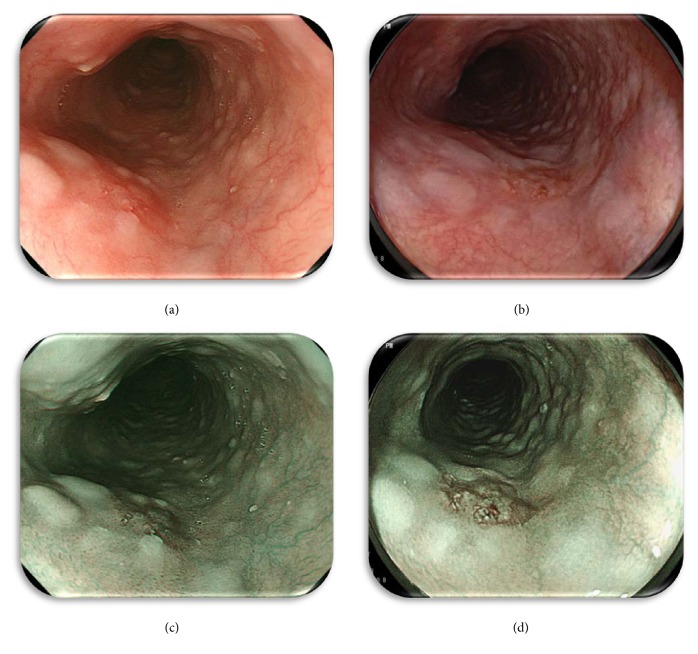
Representative still images: superficial flat depressed lesion(0-IIc type) on the middle thoracic esophagus. (a) O-WLI image, (b) F-WLI image, (c) NBI image, and (d) BLI-bright image.

For the subjective evaluation, the endoscopists provided a ranking score (RS) of the endoscopic images using the following 3-point ranking method based on ease of recognition of the cancerous area. Images with the easiest recognition were given 3 points; those with a comparatively lower degree of clarity were given 2 points, and obscure images scored only 1 point. Images obtained with each modality (O-WLI, F-WLI, NBI, and BLI-bright) were prepared for evaluation by placing them on a computer monitor and displaying them independently of the images obtained with the other endoscopic modality. A representative set of still images for the ESCC case is shown in Figure 1. The endoscopists viewed the images individually, without side-by-side comparison with other images of the same lesion obtained by the other methods. Each image was scored using the above scale with 1–3 points. The images were evaluated by three endoscopists (NY, OD, and RK). Images were displayed and observed with a personal computer in the standard and widely supported red/green/blue (RGB) mode.

### 2.6. Objective Evaluation

**Figure 2:**
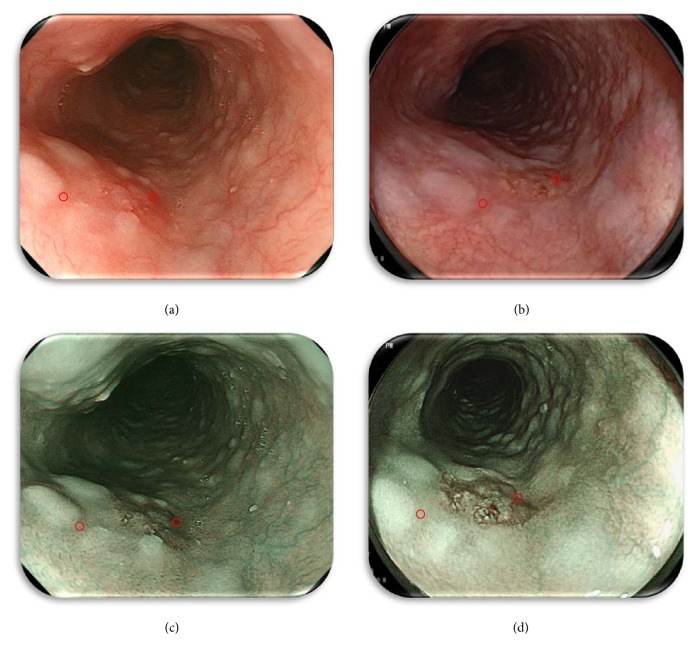
Representative still images illustrating the spots captured for the color difference score (CDS) calculation of the lesion and background mucosa (Figure 1). (a) O-WLI image, (b) F-WLI image, (c) NBI image, and (d) BLI-bright image. The lesion was captured for image processing, and the region of interest (ROI) was highlighted to calculate the CDS using each of the four methods.

**Figure 3:**
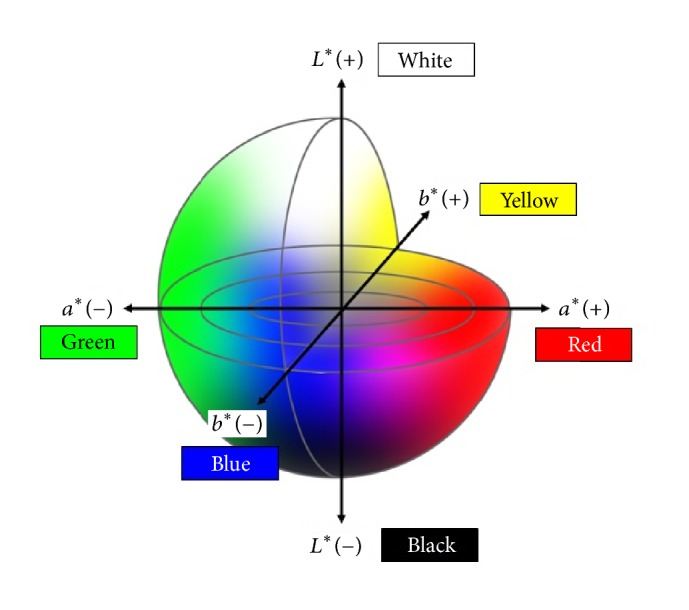
In the *L* ^*∗*^ *a* ^*∗*^ *b* ^*∗*^ color space system the color differences are visualized as distances in a diagram. *L* ^*∗*^: color brightness (*L* ^*∗*^ = 0 is black and *L* ^*∗*^ = 100 is white). *a* ^*∗*^: position between red and green (negative values are progreen; positive values are prored). *b* ^*∗*^: position between yellow and blue (negative values are problue; positive values are proyellow).

The following methods were used to ensure that the endoscopic images were objectively evaluated. Each lesion and the surrounding mucosa were captured for image processing, and the region of interest (ROI) was highlighted. Representative still images illustrating the spots captured for the color difference score (CDS) calculation of the lesion and background mucosa are shown in Figure 2. In order to ensure the accuracy of this method, the ROIs were selected under the following conditions: (1) each ROI in the set of four images was selected if located in the same area of the lesion, (2) domains with excess brightness, darkness, or particular halation were excluded, and (3) each ROI figure was square shaped with a side length of more than 20 pixels. The color difference score (CDS: Δ*E* = [(Δ*L* ^*∗*^)^2^ + (Δ*a* ^*∗*^)^2^ + (Δ*b* ^*∗*^)^2^]^1/2^) of the pixel values based on *L* ^*∗*^ *a* ^*∗*^ *b* ^*∗*^ (Lab) color spaces within the ROI was used to evaluate the recognition ability of each color image [18]. A Lab color space is a color-opponent space with three dimensions. The three dimensions are lightness (*L*), redness/greenness (*a*), and yellowness/blueness (*b*). Lab color space is based on how the human eye interprets color not how RGB or CMYK color models represent color. It is intended to provide perceptual uniformity, and its *L* component closely matches the human perception of lightness. It can thus be used to make accurate color balance corrections by modifying output curves in the a and b components or to adjust the lightness contrast by using the *L* component. Therefore, delta *E*, indicating the distance in Lab space, is suitable for estimating the difference between colors (Figure 3). The average color value in the ROI was calculated by using Photoshop CS4 image-editing software (Adobe Systems Inc.) as follows: (1) the image was opened, (2) the ROI was placed manually by using the Rectangular Marquee mode to locate the same area in the corresponding images, (3) the color mode was transformed from RGB to Lab, (4) the average of the color values (*L*, *a*, *b*) in the ROI was determined from the Histogram panel, and (5) these color values were converted into the units of CIE LAB. The conversion utilized the following formula:

(1) L∗=L256×100,a∗=a−128,b∗=b−128,

where (*L*, *a*, *b*) are the color values determined with Photoshop and (*L* ^*∗*^, *a* ^*∗*^, *b* ^*∗*^) are the color values in the CIE LAB unit [19].

### 2.7. Statistical Analysis

A post hoc power analysis was conducted to determine the power for the sample size (*n* = 25). A post hoc power analysis was performed by using G^*∗*^Power ver 3.1 [20]. Analysis of subjective evaluation compared to the total RS average for the four different methods by three endoscopists. The Wilcoxon signed-rank test was used to analyze the diagnostic capability of the four different methods both subjectively and objectively. A *P* value of <0.05 was considered statistically significant.

Inter- and intraobserver concordance was evaluated by calculating the *κ*-value. Interobserver variation was calculated from the results of the second reading. Intraobserver variation was determined by comparing the first and second assessments for each endoscopist. A *κ*-value of >0.8 indicated excellent agreement, 0.6–0.79 indicated substantial agreement, 0.4–0.59 indicated moderate agreement, 0.2–0.39 indicated fair agreement, and <0.2 indicated slight or very poor agreement. All statistical analyses were performed using SPSS software, Version 22.0 for Windows (IBM Japan, Ltd, Tokyo, Japan).

## 3. Results

### 3.1. Histopathological Findings

The clinicopathological features of the patients are shown in Table 1. 25 patients (20 men, 5 women; age range, 55–87 y) were enrolled in the study. The mean size of the ESCCs was 20.5 mm (4–42 mm). Histologically, there were 24 intramucosal ESCCs and 1 ESCC with submucosal invasion. The endoscopic appearance of the ESCCs was 0-IIa type in 3 patients, 0-IIb type in 14 patients, and 0-IIc type in 8 patients.

### 3.2. Ranking Score

**Figure 4:**
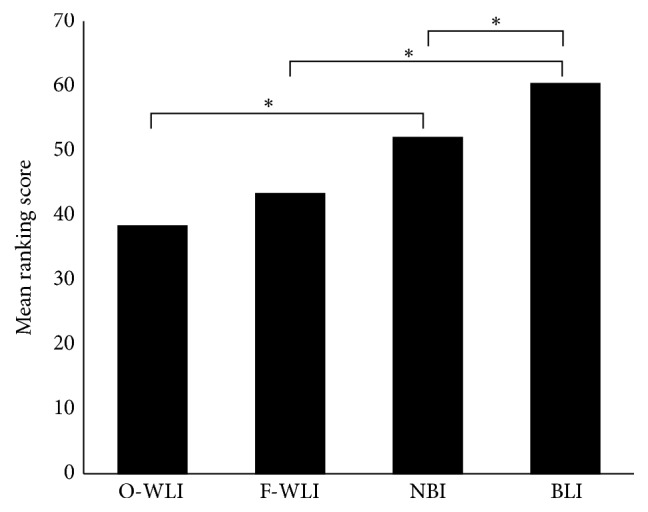
Subjective evaluation. Statistical comparison of the ranking score of O-WLI, F-WLI, NBI, and BLI-bright images for quality of ESCC visualization (Wilcoxon signed-rank test). Numbers above each row denote the numbers of ESCCs. ^*∗*^ *P* < 0.01.

The total RS of the four methods by the endoscopists was as follows: O-WLI was 35/32/48 (NY/OD/RK) (mean, 35.3), F-WLI was 37/35/58 (mean, 43.3), NBI was 48/47/61 (mean, 52.0), and BLI-bright was 54/59/68 (mean, 60.3; Figure 4). There was no significant difference between the total RS of O-WLI and F-WLI. The total RS of NBI was significantly higher than that of O-WLI (*P* < 0.01) and the total RS of BLI-bright was significantly higher than that of F-WLI (*P* < 0.01). Moreover, the total RS of BLI-bright was significantly higher than that of NBI (*P* < 0.01).

The *κ*-values for interobserver (NY, OD, and RK) agreement were as follows: NY to OD was 0.613, NY to RK was 0.382, and OD to RK was 0.317. The *κ*-values of intraobserver agreement were as follows: 0.673 (NY), 0.873 (OD), and 0.599 (RK; Table 2).

### 3.3. Color Difference Scores

**Figure 5:**
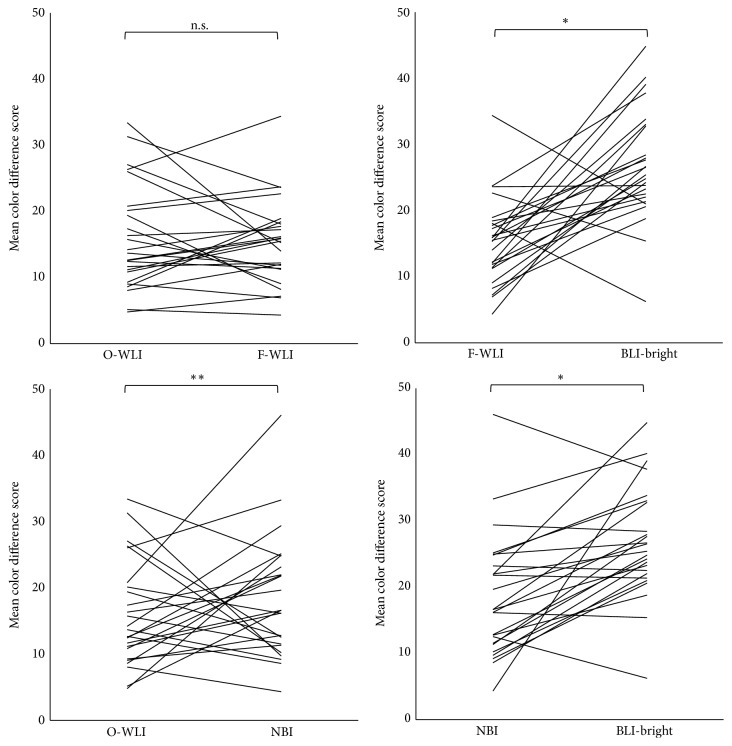
Objective evaluation. Statistical comparison of the mean CDS of O-WLI, F-WLI, NBI, and BLI-bright images for quality of ESCC visualization (Wilcoxon signed-rank test). n.s.: not significant. ^*∗*^ *P* < 0.01, ^*∗∗*^ *P* < 0.05.

The mean CDS of O-WLI, F-WLI, NBI, and BLI-bright were 15.92, 15.27, 18.43, and 26.72, respectively. There was no significant difference between the mean CDS of O-WLI and F-WLI. The mean CDS of NBI was significantly higher than that of O-WLI (*P* < 0.05), and the mean CDS of BLI-bright was significantly higher than that of F-WLI (*P* < 0.01). Moreover, the mean CDS of BLI-bright was significantly higher than that of NBI (*P* < 0.01; Figure 5).

### 3.4. Post Hoc Power Analysis

In 25 study patients, post hoc power analysis demonstrated a statistical power value greater than 90% for FWL versus BLI (power = 0.99) and BLI versus NBI (power = 0.97) for both the subjective and objective evaluation.

### 3.5. Representative Cases

These images are representative of a 0-IIc type early squamous cell carcinoma of the middle thoracic esophagus (Figure 1). A typical ESCC was observed as a reddish area by using O-WLI (Figure 1(a)) and F-WLI (Figure 1(b)) or as a brownish area by using NBI (Figure 1(c)) and BLI-bright (Figure 1(d)) in a distant view. The results of subjective evaluation by the three respective endoscopists were as follows: O-WLI images were 2 points/1 point/2 points (total RS: 5 points), F-WLI images were 2 points/1 point/1 point (total RS: 4 points), NBI images were 2 points/2 points/2 points (total RS: 6 points), and BLI-bright images were 2 points/3 points/3 points (total RS: 8 points). The results of objective assessment (CDS) of each ROI (Figure 2) were O-WLI, 26.01; F-WLI, 15.29; NBI, 33.27; and BLI-bright, 40.17.

## 4. Discussion

This is the first report of the use of BLI-bright for the detection of superficial ESCC in comparison with WLI in a distant view. Previous studies have shown that the detection rate of NBI for superficial ESCC is higher than that of WLI [13]. The results of the current study suggest that BLI-bright has a higher detection ability for superficial ESCCs than WLI. Therefore, BLI-bright might improve the detection rate of superficial ESCCs during screening endoscopy in a manner similar to NBI.

BLI-bright facilitates the detection of ESCCs that show a well-demarcated brownish area. An important element for the detection of a brownish area in a distant view is the background coloration of the epithelium between each of the intraepithelial papillary capillary loops (IPCL). Recently, it has been reported that the presence of hemoglobin in the epithelium of ESCC is an important factor affecting background coloration [21]. BLI-bright light is composed of two specific wavelengths that are strongly absorbed by hemoglobin. Therefore, it is possible that BLI-bright detects ESCC as a well-demarcated brownish area as well as in detection with NBI.

We compared the endoscopic recognition of superficial ESCCs using four different methods (OWL, FWL, NBI, and BLI-bright). Our study showed the utility of IEE compared with WLI by both RS and CDS. Furthermore, we also showed the utility of BLI-bright compared with NBI by both RS and CDS. In this way, lesions detected with BLI-bright were significantly more visible than those detected with the other methods, both subjectively and objectively. Additionally, the differences between the Olympus and Fujifilm system specifications, such as field of view and depth of field, were negligible and did not influence the results of our study.

The *κ*-value of interobserver agreement for subjective evaluations was fair to substantial (0.317–0.613). One endoscopist (RK) had a high evaluation standard with WLI, decreasing the *κ*-value. The *κ*-value for intraobserver agreement was moderate to excellent (0.599–0.873). Post hoc power analysis indicated that our research had sufficient to moderate power to detect significant group differences for BLI, FWL, and NBI [20].

Our study has three major limitations. First, we evaluated NBI images with a first-generation NBI system in this study. Since BLI-bright provides a brighter image than the first generation of NBI, it is possible that this contributed to the higher detection ability in the distant view. Second, the number of cases was small, and the data were gathered from a single center. Third, we evaluated only still images. Therefore, further multi-institutional studies with a larger number of cases are required to compare real-time images for ESCC detection with WLI, BLI-bright, and NBI.

## 5. Conclusion

In conclusion, BLI-bright visualized superficial ESCC better than the other methods tested (O-WLI, F-WLI, and NBI), both subjectively and objectively. BLI-bright may be a useful tool for detecting superficial ESCCs during screening endoscopy.

## Figures and Tables

**Table 1:** Clinicopathological features of patients.

Patients/lesions	25/25
Median age, years (range)	70 (55–87)
Sex	
Male	20
Female	5
Mean tumor size, mm (range)	20.5 (4–42)
Depth	
Intramucosal	24
Submucosal	1
Macroscopic type	
0-IIa type	3
0-IIb type	14
0-IIc type	8

**Table 2:** Inter- and intraobserver agreement (*κ*-value) for ranking score.

	NY to OD	NY to RK	OD to RK
Interobserver agreement	0.613	0.382	0.317

	NY	OD	RK

Intraobserver agreement	0.673	0.873	0.599
